# 两性霉素B脂质体挽救性治疗80例血液病患者侵袭性真菌病临床疗效分析

**DOI:** 10.3760/cma.j.cn121090-20240228-00075

**Published:** 2024-07

**Authors:** 源兵 吴, 珊珊 姜, 雅雪 吴, 彬 刘, 雨童 景, 海燕 包, 骁 马, 德沛 吴, 晓慧 胡

**Affiliations:** 1 苏州弘慈血液病医院血液科，苏州 215000 Department of Hematology, The Hospital of Suzhou Hongci Hematology, Suzhou 215000, China; 2 苏州大学附属第一医院血液科，江苏省血液病研究所，国家血液系统疾病临床医学研究中心，苏州 215000 Department of Hematology, The First Affiliated Hospital of Soochow University, Jiangsu Institute of Hematology, National Clinical Research Center for Hematologic Diseases, Suzhou 215000, China

**Keywords:** 两性霉素B脂质体, 血液病, 侵袭性真菌病, Liposomal amphotericin B, Hematological diseases, Invasive fungal disease

## Abstract

**目的:**

探究两性霉素B脂质体（Liposomal amphotericin B，L-AmB）在血液病患者中挽救性治疗侵袭性真菌病（invasive fungal disease，IFD）的疗效及安全性。

**方法:**

回顾性收集既往抗真菌治疗失败后2023年6月至2023年12月期间于苏州弘慈血液病医院血液科接受L-AmB治疗的80例血液病患者资料。统计患者基本信息、临床疗效，应用Logistic回归分析影响L-AmB疗效的因素。

**结果:**

80例血液病患者中，确诊IFD 9例（11.2％），临床诊断IFD 43例（53.8％），拟诊IFD 28例（35.0％）。L-AmB挽救性治疗有效率为77.5％，中位每日剂量3（1～5）mg·kg^−1^·d^−1^，中位用药疗程14（8，25）d。多因素Logistic回归分析显示：疾病缓解状态（*OR*＝4.337，95％ *CI* 1.167～16.122，*P*＝0.029）和用药疗程（*OR*＝1.127，95％ *CI* 1.029～1.234，*P*＝0.010）是影响L-AmB疗效的独立因素。L-AmB相关输液反应包括发热和寒战（5.0％）。低钾血症发生率为28.8％，主要为1～2级。肾毒性发生率为11.3％，主要为1～2级。

**结论:**

L-AmB治疗既往抗真菌治疗不耐受或无效的IFD患者安全有效，不良反应率低。

## Abstract (en)

**Objective:**

To investigate the efficacy and safety of liposomal amphotericin B（L-AmB）for the salvage treatment of invasive fungal disease（IFD）in patients with hematological diseases.

**Methods:**

Data were retrospectively collected from 80 patients with hematological issues treated with L-AmB between June 2023 and December 2023 after failure of previous antifungal therapy. Baseline patient information, clinical efficacy, and factors affecting the efficacy of L-AmB were analyzed by logistic regression. Moreover, adverse effects associated with L-AmB were evaluated.

**Results:**

Among the 80 patients, 9（11.2％）had proven IFD, 43（53.8％）had probable IFD, and 28（35.0％）had possible IFD. The efficacy rate of L-AmB salvage therapy for IFD was 77.5％, with a median daily dose of 3（range: 1–5）mg·kg^−1^·d^−1^ and a median dosing course of 14（range: 8–25）days. Multivariate logistic regression analysis showed that the disease remission status（*OR*＝4.337, 95％ *CI* 1.167–16.122, *P*＝0.029）and duration of medication（*OR*＝1.127, 95％ *CI* 1.029–1.234, *P*＝0.010）were independent factors affecting the efficacy of L-AmB. The incidence of infusion reactions associated with L-AmB, including fever and chills, was 5.0％. The incidence of hypokalemia was 28.8％（predominantly grades 1–2）, and the incidence of nephrotoxicity was 11.3％（predominantly grades 1–2）.

**Conclusion:**

L-AmB is safe and effective in the treatment of patients with IFD who are intolerant to or who have experienced no effect of previous antifungal therapy, with a low rate of adverse reactions.

侵袭性真菌病（IFD）是指真菌病原体侵入人体导致的感染性疾病，主要发生在免疫力低下的人群。近年来，IFD的发病率呈逐年上升趋势[1]。我国血液系统恶性肿瘤患者确诊和临床诊断IFD发生率在接受化疗人群中为2.1％，在接受造血干细胞移植（Hematopoietic stem cell transplantation，HSCT）人群中达7.7％[2]。IFD是血液系统恶性肿瘤患者重要死亡原因之一[3]，HSCT后IFD相关病死率高达50％，肺部是IFD主要侵犯部位[4]。曲霉与念珠菌是血液病患者IFD最常见致病菌，毛霉则相对少见。近年来随着三唑类抗真菌药预防治疗IFD的开展，多重耐药及罕见真菌感染呈上升趋势[5]，血液疾病患者IFD的治疗面临新的挑战。

普通两性霉素B（Amphotericin B，AmB）具有强效广谱的抗真菌活性，特别是抗耐药真菌感染，但由于其毒副作用明显，影响其临床广泛应用。AmB主要剂型有两性霉素B脱氧胆酸盐（Amphotericin B deoxycholate，AmBD）和两性霉素B脂质制剂：包括脂质体（L-AmB）、脂质复合物（ABLC）和胶体分散体（ABCD，即硫酸胆固醇酯）3种剂型。L-AmB是一种小球形脂质体结构，其主要成分为氢化大豆磷脂酰胆碱、二硬脂酰磷脂酰甘油、胆固醇等，各种结构与两性霉素B分子紧密结合，进入体内后仍能以脂质体形态保留，药物清除时间长，所以相对于AmBD，L-AmB对肾功能影响较小[6]。L-AmB与AmBD抗真菌活性相似，但L-AmB引起的肾损伤以及输液反应发生率更低，提示L-AmB安全性优于AmBD[7]。

L-AmB于2023年6月在国内上市，适用于深部真菌感染的患者，肾损伤或药物毒性而不能使用有效剂量AmB患者，或已经接受过AmB治疗无效的患者。目前关于此药在国内临床使用数据较少，因此，本文回顾性分析既往抗真菌治疗失败后使用L-AmB治疗IFD的血液病患者的临床资料，评价L-AmB治疗IFD的疗效及安全性，为临床用药提供数据支持。

## 病例与方法

一、研究对象

回顾性收集2023年6月至2023年12月期间在苏州弘慈血液病医院诊断为IFD且接受L-AmB治疗的80例血液病患者临床资料。纳入标准：①血液系统疾病患者且接受化疗或HSCT；②IFD诊断标准依据《血液病/恶性肿瘤患者IFD的诊断标准与治疗原则（第六次修订版）》[2]；③既往接受抗真菌治疗无效或药物不耐受后使用L-AmB挽救性治疗的IFD患者（包括确诊、临床诊断、拟诊IFD）；④临床资料完整。排除标准：①患者年龄<18岁；②接受L-AmB治疗<3 d。

收集数据包括：患者年龄、性别、疾病类型及疾病缓解状态；既往使用抗真菌药物及疗效评估、抗真菌治疗时间和药物类型；L-AmB不良反应。微生物学证据源于血液培养、肺泡灌洗液培养、痰培养、深部感染部位的病理活检、病原体宏基因组二代测序、G试验、GM试验、影像学检查。仅评估第1次使用L-AmB情况。

二、评价指标

完全缓解：患者在观察期内生存，IFD相关症状和体征、影像学异常全部消失，微生物学证据提示真菌清除。部分缓解：患者在观察期内生存，IFD相关症状和体征、影像学异常有所好转，微生物学证据和实验室标志物检测提示，真菌负荷减轻或清除的证据。完全缓解和部分缓解被认定为临床有效。稳定：患者在观察期内生存，根据临床、影像学和真菌学综合评估提示，IFD轻微或没有改善，但没有进展的证据。疾病进展：基于临床、影像学和微生物学综合评估证实IFD进展。死亡：在评估期间，因任何原因死亡。稳定、疾病进展、死亡被认定为临床无效。不良反应评估：根据常见不良反应事件评价标准（Common Terminology Criteria for Adverse Events，CTCAE）V5.0来评估患者不良反应。

三、治疗方案

注射用两性霉素B脂质体（国药准字HJ20233123，规格50 mg/支）。既往使用的三唑类抗真菌药物为伊曲康唑、泊沙康唑、伏立康唑以及艾沙康唑，棘白霉素类抗真菌药为卡泊芬净，多烯类抗真菌药物为普通两性霉素B。药物使用后按照《血液病/恶性肿瘤患者IFD的诊断标准与治疗原则（第六次修订版）》[2]进行评估，经评估患者出现不耐受副作用或者治疗无效，换用L-AmB进行挽救性治疗。部分患者使用L-AmB前予糖皮质激素预处理预防输液反应。

四、统计学处理

采用SPSS 22.0软件进行数据分析。计量资料用中位数（*P*25，*P*75）进行统计描述，采用秩和检验进行组间比较。计数资料采用例数（百分比）进行统计描述，采用*χ*^2^检验或Fisher切确概率法进行组间比较。双侧*P*<0.05为差异有统计学意义。

## 结果

一、患者一般特征

共80例患者纳入研究，中位年龄为49（35，60）岁，52例（65.0％）为男性。基础疾病包括急性髓系白血病36例（45.0％）、急性淋巴细胞白血病10例（12.5％）、再生障碍性贫血7例（8.8％）、骨髓增生异常综合征15例（18.8％）等。既往使用的抗真菌治疗药物包括三唑类46例（57.5％）、棘白菌素类卡泊芬净10例（12.5％）、多烯类普通两性霉素B 8例（10.0％）以及这些药物的联合使用。20.0％（16/80）患者换用L-AmB前对既往抗真菌治疗的药物不耐受，64.0％（64/80）患者对既往抗真菌药物无效。最主要的感染部位为肺部88.8％（71/80）、其次是血流11.2％（9/80）。最主要的感染病原菌为曲霉属22.5％（18/80），其次为念珠菌属21.3％（17/80）、毛霉属6.3％（5/80）。本次IFD诊断分层为拟诊IFD 28例（35.0％）、临床诊断IFD 43例（53.8％）、确诊IFD 9例（11.2％）（表1）。

**表1:** 80例IFD患者接受两性霉素B脂质体（L-AmB）治疗的临床特征

临床特征	值
年龄[岁，*M*（*P*_25_, *P*_75_）]	49（35, 60）
体重[kg，*M*（*P*_25_, *P*_75_）]	59.0（52.0, 65.8）
性别[例（%）]	
女	28（35.0）
男	52（65.0）
血液病类型[例（%）]	
AML	36（45.0）
ALL	10（12.5）
AA	7（8.8）
MDS	15（18.8）
其他	12（15.0）
前期抗真菌药物[例（%）]	
三唑类	46（57.5）
棘白霉素类	10（12.5）
多烯类	8（10.0）
棘白霉素类+多烯类	2（2.5）
三唑类+棘白霉素类	12（15.0）
三唑类+多烯类	2（2.5）
前期抗真菌治疗失败原因[例（%）]	
不耐受	16（20.0）
无效	64（80.0）
感染部位[例（%）]	
肺部	71（88.8）
血流	9（11.2）
鼻窦、颅内	4（5.0）
≥2个部位	15（18.8）
病原体[例（%）]	
毛霉菌属	5（6.3）
念珠菌属	17（21.3）
曲霉菌属	18（22.5）
不明病原体	40（50.0）
诊断分层[例（%）]	
拟诊	28（35.0）
临床诊断	43（53.8）
确诊	9（11.2）
治疗分层[例（%）]	
诊断驱动治疗	28（35.0）
目标治疗	52（65.0）
糖皮质激素预处理[例（%）]	
是	23（28.8）
否	57（71.2）
L-AmB剂量[mg·kg^−1^·d^−1^，*M*（范围）]	3（1~5）
用药疗程[d，*M*（*P*_25_, *P*_75_）]	14（8, 25）
累积剂量[mg，*M*（*P*_25_, *P*_75_）]	1800（1050, 3388）
L-AmB退热时间[d，*M*（*P*_25_, *P*_75_）]	3（2, 5）
肺部病灶缩小时间[d，*M*（*P*_25_, *P*_75_）]	17.5（11, 27）
治疗反应[例（%）]	
完全缓解	21（26.2）
部分缓解	41（51.3）
稳定	8（10.0）
进展	6（7.5）
死亡	4（5.0）

**注** IFD：侵袭性真菌病；AML：急性髓系白血病；ALL：急性淋巴细胞白血病；MDS：骨髓增生异常综合征；AA：再生障碍性贫血

二、疗效分析

使用L-AmB对这80例IFD血液病患者进行治疗，其中28例（35.0％）为诊断驱动治疗，52例（65.0％）为目标治疗。在L-AmB使用前23例（28.8％）患者使用激素预处理，57例（71.2％）患者未进行预处理。L-AmB中位每日剂量为3（1～5）mg·kg^−1^·d^−1^，中位用药疗程为14（8，25）d，中位累积剂量1800（1050，3388）mg。使用L-AmB后，中位退热时间为3（2，5）d，肺部病灶缩小的中位时间为17.5（11，27）d。根据疗效评价标准：L-AmB治疗后26.2％（21/80）患者获得完全缓解，51.3％（41/80）患者获得部分缓解，10.0％（8/80）稳定，7.5％（6/80）进展，5.0％（4/80）死亡，临床有效率为77.5％（62/80）（表1）。

单因素分析显示，患者年龄<60岁、血液疾病处于缓解状态，L-AmB每日剂量≥3.0 mg·kg^−1^·d^−1^、用药疗程≥14 d，L-AmB治疗IFD临床疗效更好（均*P*<0.05）（表2）。此外，L-AmB单药治疗对比L-AmB联合治疗IFD感染的完全缓解率（33.3％对19.5％，*P*＝0.283）及部分缓解率（41.0％对61.0％，*P*＝0.309）差异无统计学意义，同时临床中位退热时间［3（2.0, 6.5）d对3（2.8, 4.0）d，*P*＝0.606］及肺部感染病灶中位缩小时间［23（11, 30）d对14（9, 24）d，*P*＝0.203］差异无统计学意义。

**表2:** 影响两性霉素B脂质体治疗IFD疗效的单因素分析

变量	例数	临床有效组 （62例）	临床无效组 （18例）	*P*值
年龄<60岁[例（%）]	56	47（83.9）	9（16.1）	0.035
男性[例（%）]	52	39（75.0）	13（25.0）	0.466
血液病类型[例（%）]				0.124
AML	36	31（86.1）	5（13.9）	
ALL	10	8（80.0）	2（20.0）	
AA	7	3（42.9）	4（57.1）	
MDS	15	12（80.0）	3（20.0）	
其他	12	8（66.7）	4（33.3）	
原发病未缓解[例（%）]	34	21（61.8）	13（38.2）	0.004
未接受造血干细胞移植[例（%）]	28	20（71.4）	8（28.6）	0.340
中性粒细胞缺乏[例（%）]	25	19（76.0）	6（24.0）	0.829
粒缺持续时间≥10 d[例（%）]	22	16（72.7）	6（27.3）	0.352
IFD诊断分层[例（%）]				0.340
拟诊	28	20（71.4）	8（28.6）	
临床诊断或确诊	52	42（80.8）	10（19.2）	
感染部位[例（%）]				0.452
肺部	71	55（77.5）	16（22.5）	
血流	9	7（77.8）	2（22.2）	
鼻窦、颅内	4	2（50.0）	2（50.0）	
病原体[例（%）]				0.466
毛霉菌属	5	3（60.0）	2（40.0）	
念珠菌属	17	14（82.4）	3（17.6）	
曲霉菌属	18	12（66.7）	6（33.3）	
每日剂量≥3.0 mg·kg^−1^·d^−1^[例（%）]	41	36（87.8）	5（12.2）	0.024
用药疗程≥14 d[例（%）]	41	39（95.1）	2（4.9）	<0.001
两性霉素B脂质体治疗方案[例（%）]				0.512
单药治疗	39	29（74.4）	10（25.6）	
联合治疗	41	33（80.5）	8（19.5）	

**注** IFD：侵袭性真菌病；AML：急性髓系白血病；ALL：急性淋巴细胞白血病；MDS：骨髓增生异常综合征；AA：再生障碍性贫血

将单因素分析中*P*<0.05的变量纳入多因素Logistic回归，结果显示L-AmB用药疗程（*OR*＝1.127，95％ *CI* 1.029～1.234，*P*＝0.010）以及L-AmB治疗IFD时疾病是否缓解（*OR*＝4.337，*CI* 1.167～16.122，*P*＝0.029）是影响L-AmB疗效的独立因素，而L-AmB的每日剂量及患者年龄并不影响L-AmB疗效（表3）。

**表3:** 多因素Logistic回归分析影响两性霉素B脂质体治疗侵袭性真菌病（IFD）疗效的因素

变量	*OR*	95% *CI*	*P*值
每日剂量≥3.0 mg·kg^−1^·d^−1^	1.252	0.337~4.642	0.737
年龄≥60岁	1.078	0.784~1.483	0.645
血液病状态（未缓解，缓解）	4.337	1.167~16.122	0.029
用药疗程（<14 d，≥14 d）	1.127	1.029~1.234	0.010

三、安全性

L-AmB治疗过程中有4例（5.0％）患者出现输液相关反应（发热和寒战），3例（3.8％）出现恶心及呕吐，经对症处理后症状迅速消失。低血钾发生率为28.8％（23/80），主要为1～2级，经加强补钾治疗后血钾恢复正常。有2例（2.5％）患者出现顽固性低钾血症，其中1例为3级低钾血症，经停止用药3 d及补钾治疗后血钾恢复正常；另1例为4级低钾血症，经停止用药5 d及补钾治疗后血钾恢复正常。低镁血症的发生率为20.0％（16/80），主要为1～2级，经补镁治疗后恢复正常。80例患者中有9例（11.3％）出现肾毒性，主要为1～2级。经L-AmB减量、加强保肾治疗5～7 d后肾功能恢复正常。此外，研究结果表明L-AmB的每日剂量、用药疗程以及累积剂量与肾毒性的发生差异均无统计学意义（均*P*>0.05）（表4）。

**表4:** 两性霉素B脂质体治疗侵袭性真菌病（IFD）的肾毒性发生情况

变量	有肾毒性（9例）	无肾毒性（71例）	*P*值
每日剂量≥3.0 mg·kg^−1^·d^−1^[例（%）]	6（66.7）	35（49.3）	0.326
用药疗程[d，*M*（*P*_25_, *P*_75_）]	9（11，21）	8（15，26）	0.477
累积剂量[mg，*M*（*P*_25_, *P*_75_）]	1525（1 700，4 275）	1000（1 800，3 400）	0.848

## 讨论

IFD是血液病患者感染性致死病因之一，有效的抗真菌治疗对改善患者预后至关重要。与AmBD相比，L-AmB在肾脏毒性、输液相关反应等安全性方面有显著改善。与唑类相比，L-AmB与血液病患者常用药物相互作用少。对唑类耐药曲霉、毛霉病患者，L-AmB是更好的治疗选择。

本研究以80例IFD感染的血液病患者为研究对象，经L-AmB挽救性治疗有62例（77.5％）获得很好的临床疗效，与既往文献报道L-AmB治疗国外人群中IFD感染有效率（50％～89％）结果一致[8]–[11]。本研究中IFD主要发生在急性髓系白血病（45.0％）及骨髓增生异常综合征（18.8％），主要的发生部位为肺部（88.8％）及血流（11.2％），主要的真菌分布为曲霉菌属，其次为念珠菌属，毛霉菌少见。这与既往前瞻性、多中心流行病学研究结果相符[4]。

德国一项随机多中心前瞻性研究显示，55例HSCT后粒细胞缺乏伴发热临床诊断或确诊的IFD患者，经L-AmB单药或者联合卡泊芬净治疗14 d，两组患者之间疗效及生存率无明显差异[12]。我们的研究结果与此研究结果相一致，L-AmB联合用药疗效并不优于单药治疗。一项Ⅱ期临床试验显示L-AmB 1.0 mg·kg^−1^·d^−1^经验性治疗血液肿瘤患者中性粒细胞减少伴持续性发热获得66.3％有效率[13]。另一项纳入201例IFD患者随机接受14 d L-AmB 3 mg·kg^−1^·d^−1^或10 mg·kg^−1^·d^−1^治疗后转为L-AmB 3 mg·kg^−1^·d^−1^治疗，两组患者有效率分别为50％和46％[8]。此项研究表明使用L-AmB剂量≥3.0 mg·kg^−1^·d^−1^疗效优于<3.0 mg·kg^−1^·d^−1^。对于侵袭性念珠菌感染，欧洲临床微生物和感染性疾病学会（European Society of Clinical Microbiology and Infectious Diseases，ESCMID）推荐L-AmB剂量为3 mg·kg^−1^·d^−1^［14]、美国感染性疾病学会（Infectious Diseases Society of America，IDSA）推荐L-AmB剂量为3～5 mg·kg^−1^·d^−1^［15]。而对于毛霉病，推荐L-AmB 5～10 mg·kg^−1^·d^−1^。此外，患者年龄<60岁L-AmB治疗IFD感染疗效优于≥60岁。当血液疾病处于缓解状态，使用L-AmB治疗IFD有效率高于疾病未缓解状态。与临床无效组相比，临床有效组L-AmB中位用药时间更长，疗效更好。

本研究中L-AmB不良反应发生率低，其中治疗相关输液反应，包括发热和寒战（5.0％）。日本一项前瞻性观察研究发现，成年中性粒细胞减少伴持续发热的患者接受L-AmB治疗后输液相关反应发生率为8％[16]。我们的结果与此项研究结果相当，并且输液不良反应的发生率低于Walsh等[17]报道的结果［发热（17％）、寒战（18％）］。此外，既往随机试验研究发现L-AmB的输液相关反应明显低于AmBD（25％对63％）[18]。早期一项研究显示，大多数急性输液反应发生在输液的前5 min，而所有患者使用抗组胺药后症状迅速消退[19]。本项研究中，肾毒性的发生率为11.3％，主要为轻度肾毒性。并且肾毒性的发生和L-AmB的剂量及用药疗程无明显相关性。血液病患者在治疗过程中的时常需要联合用药，可能增加血液病患者使用L-AmB的肾毒性风险。一项意大利回顾性研究表明，血液肿瘤患者接受L-AmB联合使用环孢素A，其肾损伤发生风险是未联合使用者的2.62倍（95％ *CI* 1.10～6.27，*P*＝0.03）；若在环孢素A基础上在联合使用膦甲酸钠［次风险比（subhazard ratio，*SHR*）＝9.03, 95％ *CI* 3.68～22.14，*P*<0.001］，肾损伤风险明显上升[20]。另外，血液肿瘤及其相关并发症、放化疗均可导致肾损伤，因此血液病患者中经常出现急性肾损伤[21]。因此，治疗过程中如果出现肾功能异常，需综合考虑多方面原因，不仅仅是L-AmB所致药物性肾损伤。低血钾是L-AmB最常见的不良反应之一，此研究中，低血钾的发生率为28.8％。对于HSCT早期患者常合并存在黏膜炎，进食量少，存在钾摄入量不足，增加低血钾风险，所以此研究中低血钾的发生率偏高，可能受此因素的影响。日本一项78例首次接受L-AmB治疗的血液病患者回顾性研究显示L-AmB治疗起始2 d内开始加用钾补充剂可降低L-AmB相关低钾血症发生风险[22]。

本研究为回顾性、单中心研究，样本量较小且挽救治疗不能排除原用抗真菌药作用，具有局限性。未来需要多中心随机对照研究来进一步评估L-AmB的疗效与安全性。此外，本研究我们未研究药物不良反应发生率与药物剂量、用药时间以及其他因素的关系。

总之，本研究初步探讨了L-AmB对既往抗真菌治疗不耐受或无效的血液病患者IFD临床疗效与安全性，发现L-AmB在中国人群中具有很好的抗真菌疗效，且用药疗程及疾病缓解状态是影响其临床疗效的独立因素，治疗过程中不良反应发生率低。本研究可为临床合理使用L-AmB提供参考。
